# Rituximab therapy in patients with treatment-refractory hypersensitivity pneumonitis

**DOI:** 10.1038/s41598-025-21463-y

**Published:** 2025-10-17

**Authors:** Yosri Akl, Safy Zahid Kaddah, Shereen Medhat Nassar, Rana Kamal EL Said

**Affiliations:** Chest Department, Kasr Alainy, Faculty of Medicine, Cairo University, Giza, Egypt

**Keywords:** Hypersensitivity pneumonitis, Rituximab, FVC, Treatment, Diseases, Immunology, Medical research

## Abstract

Hypersensitivity pneumonitis (HP) presents with a highly variable clinical course, and traditional treatment includes systemic corticosteroids and strict antigen avoidance. The objective of this study was to evaluate the therapeutic potential of Rituximab in patients with progressive hypersensitivity pneumonitis who do not respond to antigen avoidance and conventional immunosuppressive therapy. In this cohort prospective study, 30 patients with refractory HP were enrolled. Forced Vital Capacity (FVC) was assessed at three time points: six months before Rituximab initiation (M − 6), at treatment initiation (M0), and six months later (M + 6). FVC% predicted significantly declined from (58.57 ± 10.9) at M − 6 to (51.03 ± 11.06%) at M0 [*p* < 0.001], but showed a minimal, non-significant drop to 50.1 ± 12.7% at M + 6 [*p* = 0.405]. The rate of decline slowed markedly post-Rituximab, from − 7.54% (M − 6 to M0) to − 0.93% (M0 to M + 6). Dyspnea, measured by the mMRC scale, improved from 2.77 ± 0.5 at M0 to 1.83 ± 0.85 at M + 6 [*p* = 0.001]. 6MWD also increased from 258.67 ± 77 m to 307 ± 99 m [*p* = 0.001]. There were no reported cases of mortality. Rituximab demonstrates potential as an emerging therapeutic option for patients with treatment-refractory hypersensitivity pneumonitis and is well-tolerated.

*Trial registration*: Retrospectively registered, registration number is NCT07035561, date of registration 16/06/2025.

## Introduction

Hypersensitivity pneumonitis (HP) is a complex interstitial lung disease characterized by inflammation and/or fibrosis of the lung parenchyma and small airways. It typically arises from an immune-mediated response to an identifiable or hidden inhaled antigen in genetically or environmentally susceptible individuals^1^. Although the precise pathophysiology remains incompletely understood, both type III (immune complex-mediated) and type IV (delayed-type) hypersensitivity reactions are thought to contribute to the development of the disease. An interplay between genetic predisposition and environmental exposures can provoke an exaggerated immune response, leading to pulmonary inflammation and, in some cases, fibrosis^2^. Anti-inflammatory and immunosuppressive agents are widely used in the treatment of various interstitial lung diseases (ILDs), including connective tissue disease-associated ILD (CTD-ILD), HP, and non-specific interstitial pneumonia (NSIP). However, when ILD progresses despite standard immunosuppressive therapy, the prognosis tends to be poor. In recent years, accumulating evidence has suggested that Rituximab may serve as a potential rescue therapy in non-idiopathic pulmonary fibrosis (non-IPF) ILDs that are refractory to conventional treatment^3^. Rituximab is a chimeric monoclonal antibody targeting the CD20 antigen found on pre-B and mature B lymphocytes, leading to peripheral B-cell depletion for an extended period, typically lasting 6 to 9 months^4^. Currently, there is no established therapeutic option for patients with HP who experience ongoing respiratory decline despite adherence to antigen avoidance strategies. This prompted our investigation into the potential clinical benefits of Rituximab in patients with HP who have not responded adequately to standard treatments, including corticosteroids and environmental antigen avoidance. This study aimed to assess the potential of Rituximab as an alternative therapeutic option for individuals with hypersensitivity pneumonitis unresponsive to conventional treatment approaches.

## Patients and methods

The present cohort prospective study was conducted at the Chest Department, Faculty of Medicine, Cairo University during the period between June 2023 to January 2025. The study was conducted in accordance with the national guidelines and regulations after receiving approval from the Research Ethics Committee of Cairo University code (MD-180–2023). The study included 30 patients diagnosed with hypersensitivity pneumonitis at the Chest Department, Kasr Al Ainy Hospital, Cairo University. Participants were recruited from both the inpatient wards and the interstitial lung disease (ILD) outpatient clinic. An informed consent was obtained from all patients participating in this study.

**Inclusion criteria**:

1. Participants aged 18 years and older.
2. Patients who were diagnosed with chronic hypersensitivity pneumonitis, confirmed through a multidisciplinary team discussion based on criteria adapted from the CHEST guidelines^5^. Eligible participants demonstrated a decline in forced vital capacity (FVC) of ≥ 5% despite receiving at least six months of treatment, which included antigen avoidance and immunosuppressive therapy such as corticosteroids and azathioprine.

**Exclusion criteria**:

1. Hypersensitivity to Rituximab.
2. Severe heart failure.
3. Moderate or severe pulmonary hypertension.
4. Fibrotic hypersensitivity pneumonitis.
5. Pregnancy.
6. Active infection.

### Data collection

Demographic data of all patients, smoking status, environmental or occupational exposure, onset and duration of symptoms, detailed history of previously received treatment, and oxygen therapy were collected. Assessment of Dyspnea grades using the modified Medical Research Council (mMRC) scale^6^, full clinical examination including assessment of vital signs and chest auscultation were assessed. Routine laboratory investigations (CBC, Coagulation profile, Liver, Kidney functions, ABG) and serum Immunoglobulins levels before and after initiation of therapy with Rituximab were obtained. All patients were screened for HBV, HCV and HIV infections by measuring hepatitis B surface antigen (HBsAg) and hepatitis B core antibody (anti-HBc), Anti-HCV Antibodies, and Anti-HIV Antibodies. Also, all patients were screened for TB infection using QuantiFERON-TB Gold test. Fiberoptic bronchoscopy with BAL cellular analysis together with HRCT were performed to establish the diagnosis of HP and classify the patients into two groups (fibrotic and non-fibrotic). Spirometry and 6 min walk test (6 MWT) were performed to evaluate pulmonary function. Spirometry was performed for each patient three times during the study period, first time was 6 months before immunosuppressive therapy and antigen avoidance (M-6), Second time was at Rituximab administration (M0) and third time was 6 months after Rituximab administration (M + 6). Echocardiography was performed to all patients to exclude moderate to severe pulmonary hypertension and heart failure. Prior to Rituximab administration patients were checked for active infection, their vital signs were checked including fever and clinical signs of infection were excluded prior to administration of Rituximab and their laboratory findings including CBC and serum CRP levels were checked to be within normal levels. Two doses of Rituximab were given, the first dose at day 0 and the second dose at day 15, 1 gram of Rituximab was given in each dose by an intravenous infusion. Patients were monitored for symptoms and signs of infections.

Sample size: A previous multicentric study by Ferreira et al. (2020)^7^ reported that the median change in FVC was − 8% in the 6 months preceding Rituximab, compared with − 3% in the 6 months following initiation of Rituximab, indicating an approximate mean difference in slopes of 5% points. Since the original study did not report standard deviations, the SDs were approximated from the reported ranges using the method of Hozo et al. (2005)^8^, and the within-patient SD of the paired difference was estimated at 7.43% assuming a correlation of 0.5. Thus, the standardized effect size was calculated as:

$$dz=\frac{\Delta }{{{\sigma _{{\text{diff}}}}}}=\frac{5}{{7.43}} \approx 0.67$$

Sample size calculation was performed using G*Power 3.1 for a two-tailed paired t-test with α = 0.05 and power = 0.80. The required minimum sample size was 20 patients. To account for potential loss to follow-up (≈ 20%), the study aimed to recruit at least 25 patients.

**Analysis**: A priori: Compute required sample size.

**Input:** Tail(s) = Two.

Effect size dz = 0.672.

α err prob = 0.05.

Power (1-β err prob) = 0.8.

**Output:** Noncentrality parameter δ = 3.0052754.

Critical t = 2.0930241.

Df = 19.

Total sample size = 20.

Actual power = 0.8134076.

Eligible patients were recruited consecutively. All patients meeting the predefined inclusion and exclusion criteria who presented to the inpatient wards and the interstitial lung disease (ILD) outpatient clinic during the recruitment period were invited to participate and enrolled consecutively until the target sample size was achieved. This consecutive approach is pragmatic for a single-center study of a rare clinical subgroup and minimizes investigator selection bias while allowing transparent reporting of the screening and enrollment process.

Statistical analysis: Data were collected, tabulated, and statistically analyzed using an IBM compatible personal computer with Statistical Package for the Social Sciences (SPSS) version 26, quantitative data were presented in the form of mean, standard deviation (SD), qualitative data were presented in the form numbers (N) and percentages (%), Chi-square test (χ2) or Fisher’s Exact test were used to study association between two qualitative variables, independent and paired samples t test (t) were used for independent and dependent comparisons of quantitative variables respectively. Logistic regression was used. Results were considered statistically significant at a P value of less than 0.05.

## Results

The primary aim of our study was to explore the potential benefit of Rituximab as a therapeutic option for individuals with hypersensitivity pneumonitis who had not responded to standard treatments. Identification of predictors of response to Rituximab was considered an exploratory objective of this study, rather than a primary endpoint.

### Demographic data

**Fig. 1:**
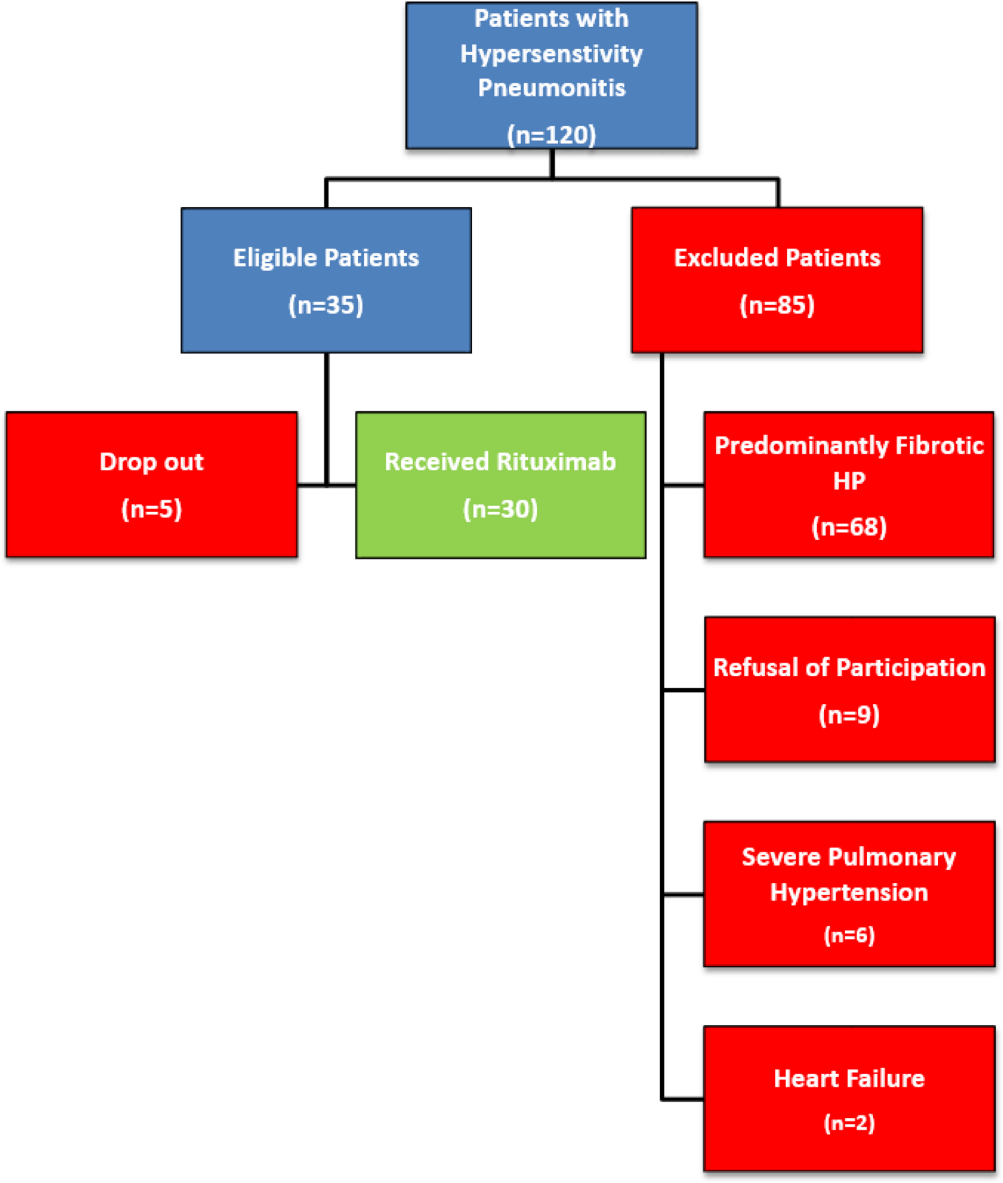
Flow diagram of the eligible and excluded patients.

A total of 120 patients were consecutively recruited for the study. Eighty-five patients were excluded for the following reasons: predominantly fibrotic hypersensitivity pneumonitis in 68 patients, refusal to participate in 9 patients, severe pulmonary hypertension in 6 patients, and heart failure in 2 patients. Among the 35 eligible patients, 5 were lost to follow-up, resulting in a final study cohort of 30 patients, as illustrated in the flowchart (Fig. 1). The study population comprised 3 male patients (10%) and 27 female patients (90%), with a mean age of 39.9 ± 10.8 years and an average body mass index (BMI) of 29.7 ± 4.6 kg/m².

The mean disease duration of hypersensitivity pneumonitis prior to initiation of rituximab therapy was 21.6 months, with a median duration of 18 months.

**Fig. 2:**
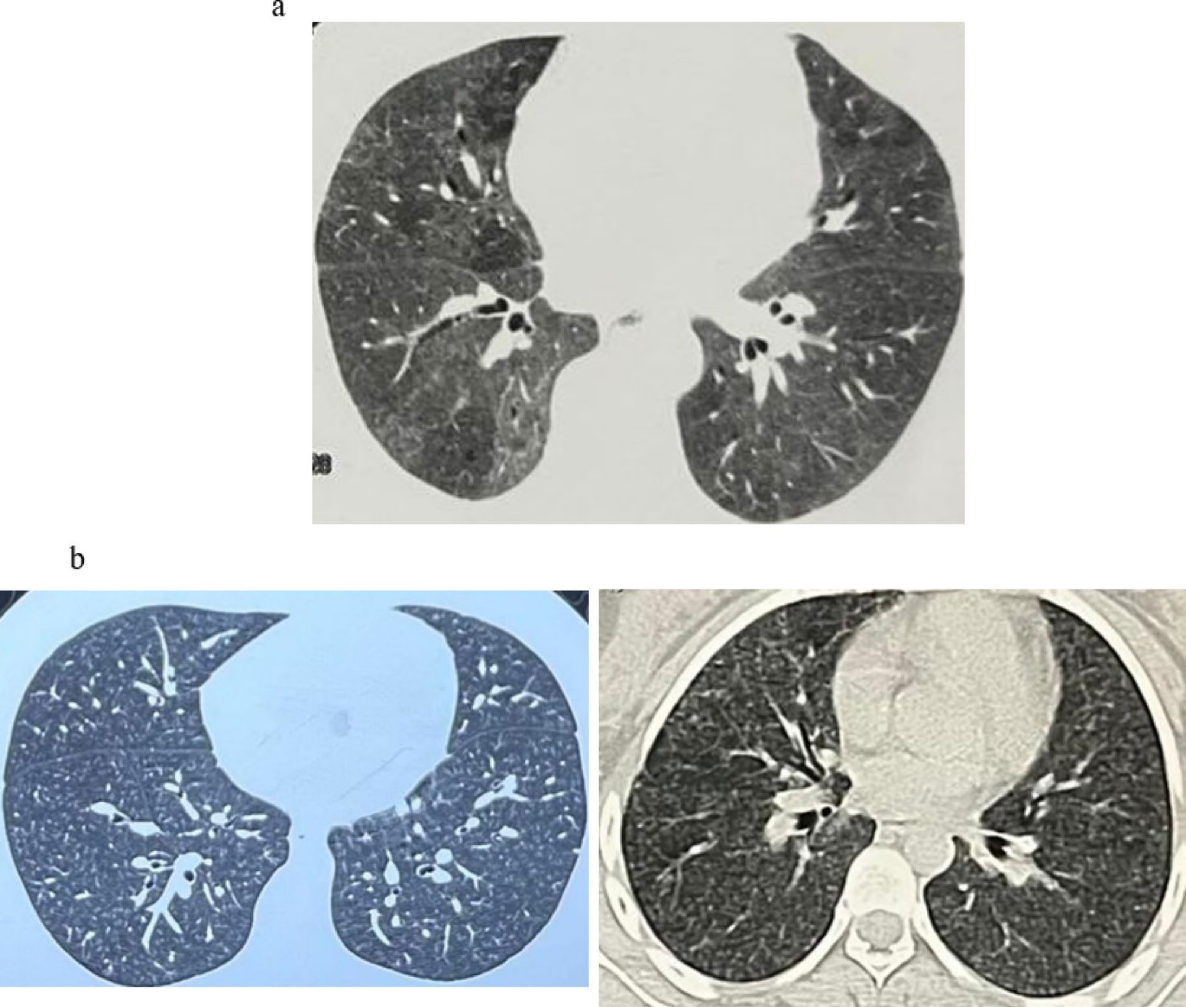
(**a**) HRCT scan of a patient from the study showing Mosaicism. (**b**) HRCT scans of patients from the study showing diffuse Centrilobular nodules.

Associated comorbidities included, 23 patients (76.7%) exhibited GERD symptoms, two patients were diabetic (6.7%) and a single case (3.3%) suffered from systemic hypertension. Patients predominantly presented with shortness of breath (96.7%), followed by cough (80%). The antigen type was identified as domestic antigen in 22 cases (73.3%) (bird breeding) and occupational antigen in a single male patient worked as a carpenter (3.3%). In 7 patients (23.3%) the antigen was not identified (unknown). The main radiological findings of our patients (HRCT) revealed centrilobular nodulations (70%) Fig. 2a, mosaicism (36.7%) Fig. 2b, and diffuse ground-glass opacities (GGOs) (30%). BAL was conducted for all patients included in the study, revealing lymphocytosis of 40% or greater in all patients, as detailed in Table 1, taking into consideration that all of our patients were predominantly non-fibrotic HP. Regarding treatment status prior to Rituximab initiation, all patients received at least six months of corticosteroid therapy, with a mean dose of 17.83 ± 4.1 mg. The median duration of corticosteroid therapy before Rituximab therapy at (M0) was 18 months. Twenty patients (66.7%) were receiving azathioprine (50 mg twice daily), and one patient (3.3%) was receiving hydroxychloroquine in addition to azathioprine. Following the initiation of rituximab, corticosteroid doses were gradually tapered to a maintenance regimen of 7.5 mg prednisolone daily, in combination with ongoing azathioprine at a dose of 50 mg twice daily. Based on our clinical experience with patients with hypersensitivity pneumonitis, a dose of 1–1.5.5 mg/kg of Azathioprine was associated with the optimal therapeutic benefit while minimizing adverse effects. Doses exceeding 1.5 mg/kg were linked to an increased incidence of adverse events, particularly liver toxicity and lack of tolerance. All patients with a documented history of antigen exposure underwent a minimum of six months of strict adherence antigen avoidance prior to the initiation of Rituximab therapy.

**Table 1:** Demographic and clinical characteristics of all studied patients at M0. (*n* = 30).

	No. %
Gender	
Male	3 (10)
Female	27 (90)
Age (mean ± SD) in years	39.9 ± 10.8
BMI (mean ± SD) in kg/m^2^	29.7 ± 4.6
Comorbidities	
HTN	1 (3.3)
DM	2 (6.7)
Hyperlipidemia	1 (3.3)
SLE	1 (3.3)
No	25 (83.3)
Smoking status	
Current	0 (0)
Former	1 (3.3)
Non-smoker	29 (96.7)
Antigen exposure	
known specific	23 (76.7)
Unknown	7 (23.3)
Antigen type	
Domestic/Birds	22 (73.3)
Occupational (carpenter)	1 (3.3)
Not known	7 (23.3)
Clinical presentation*	
SOB	29 (96.7)
Cough	24 (80)
Chest pain	2 (6.7)
GERD	23 (76.7)
HRCT Pattern*	
Centrilobular nodulations	21 (70)
Diffuse GGOs	9 (30)
Mosaicism	11 (36.7)
Thin walled cysts	1 (3.3)
Traction Bronchiectasis	6 (20)
Intralobular reticulations	5 (6.7)
Lobular distortion	1 (3.3)
Honeycombing	0 (0)
Emphysema	0 (0)
Triple density sign	4 (13.3)
Lobular air trapping	6 (20)
BAL Lymphocytosis ≥ 40%	30 (100)
Corticosteroids maintenance dose before initiation of Rituximab therapy (Prednisolone in mg)	17.83 ± 4.1
Other Immunosuppression	
Azathioprine (50 mg b.i.d)	20 (66.7)
Azathioprine, Hydroxychloroquine	1 (3.3)

*: more than one clinical presentation/HRCT Pattern could be present. BMI: body mass index. kg/m^2^: kilogram per square metre unit of surface mass density. HTN: Hypertension. DM: Diabetes Mellitus. SLE: Systemic Lupus erythematosus. HRCT: High Resolution Computed Tomography. SOB: shortness of breath. GERD: Gastro Esophageal Reflux Disease. BAL: Broncho-Alveolar Lavage. GGOs: Ground Glass Opacities. b.i.d: twice a day.

### Functional and clinical assessment before rituximab administration

At the start of Rituximab therapy, dyspnea grade according to the mMRC scale (modified Medical Research Council scale)^6^ was 2.77 ± 0.504, 6 MWD was 258.67 ± 77 m, Spo2 had changed from 94.5 ± 2.9% at rest to 83.1 ± 8.8% post 6 MWD, with desaturation change of 11.4 ± 7.04%. FVC and FEV1 values were measured as 1.73 ± 0.504 L and 1.57 ± 0.447 L respectively, also FVC% predicted and FEV1 (% predicted) were measured as 51.03 ± 11.06% and 55.2 ± 11.6% respectively; as illustrated in Table 2.

**Table 2:** Functional assessment characteristics of all studied patients at the initiation of rituximab therapy (M0) and 6 months after the initiation of rituximab therapy (M + 6).

	Mean ± SD at M0	Mean ± SD at M + 6	*P*-value
FVC (L)	1.73 ± 0.504	1.7 ± 0.52	
FVC (% predicted)	51.03 ± 11.06	50.1 ± 12.7	0.405
FEV1 (L)	1.57 ± 0.447	1.54 ± 0.44	
FEV1 (% predicted)	55.2 ± 11.6	53.7 ± 12.9	
Dyspnea grade (mMRC scale)	2.77 ± 0.504	1.83 ± 0.85	**< 0.001**
6MWD (m)	258.67 ± 77	307 ± 99	**0.001**
Resting Spo2 (%)	94.5 ± 2.9	95.9 ± 1.97	
Post 6MWT SPO2 (%)	83.1 ± 8.8	90.6 ± 8.66	
Desaturation SPO2 (%)	11.4 ± 7.04	5.33 ± 7.04	

FVC: forced vital capacity. FEV1: forced expiratory volume in one second. mMRC scale: modified Medical Research Council scale. 6MWD: 6 min walking distance. SPO2: oxygen saturation.

### Functional and clinical assessment after rituximab administration

Six months after the onset of Rituximab therapy (M + 6), dyspnea grade by mMRC scale^6^ was 1.83 ± 0.85, 6 MWD was 307 ± 99 m, Spo2 had changed from 95.9 ± 1.97% at rest to 90.6 ± 8.66% post 6 MWT, with desaturation change of 5.33 ± 7.04%. FVC and FEV1 values were measured as 1.7 ± 0.52 L and 1.54 ± 0.44 L respectively, also FVC (% predicted) and FEV1 (%predicted) were measured as 50.1 ± 12.7% and 53.7 ± 12.9% respectively; as illustrated in Table 2.

**Fig. 3:**
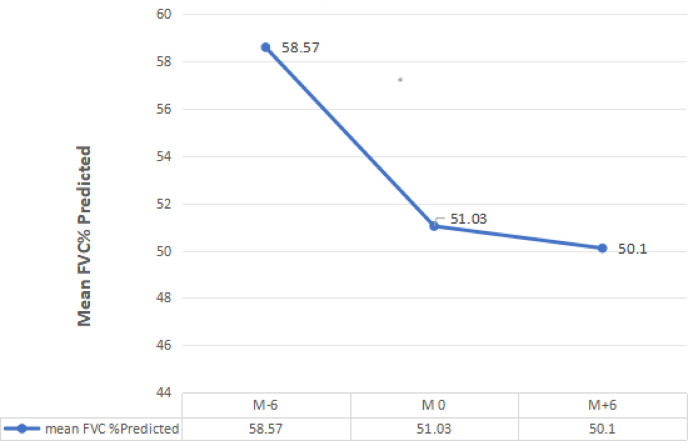
Mean FVC(% predicted) change across time points; M-6, M0 and M + 6.

Regarding the mean changes in FVC (%predicted) across treatment time points, it showed significant decrease from M-6 (6 months before Rituximab therapy initiation) (58.57 ± 10.9) to M0 (Rituximab therapy initiation) (51.03 ± 11.06), *p* < 0.001, however, there was a very minimal non-significant decrease at M + 6 (6 months after Rituximab therapy initiation) (50.1 ± 12.7) when compared with M0, *p* = 0.405. Between M0 and M + 6, FVC remained almost stable (51.03 ± 11.06 versus 50.1 ± 12.7; *p* = 0.405), as demonstrated in Fig. 3. All patients showed a decrease in FVC between M − 6 and M0; as illustrated in Table 2. Although FVC continued to decline after administration of Rituximab, the rate of decline was significantly less between M0 and M + 6 (− 0.93%) than between M − 6 and M0 (− 7.54%), *p* < 0.001, showing a rather positive response to Rituximab therapy regarding functional assessment of patients. Regarding the mean of 6 MWD, it showed significant increase from M0 (258.67 ± 77 m) to M + 6 (307 ± 99 m), *p* = 0.001; as illustrated in Table 2. Regarding dyspnea grade assessed by mMRC scale^6^, it showed a significant decrease from M 0 (2.77 ± 0.504) to M + 6 (1.83 ± 0.85), *p* < 0.001; as illustrated in Table 2.

We compared 2 groups of patients: “responders” (*n* = 16), whose FVC% of predicted at M + 6 showed a positive change; and “non-responders” (*n* = 14), whose FVC (% predicted) showed a strictly negative change compared to M0; as illustrated in Table 3.

**Table 3:** Comparison between responder and non-responder groups of patients to rituximab therapy.

		Responders (*n* = 16)	Non-responders (*n* = 14)	*P* *
RTX M0	FVC in L	1.74 ± 0.54	1.72 ± 0.48	0.915
	FVC(% predicted)	52.4 ± 11.1	49.5 ± 11.2	0.487
	FEV1 in L	1.58 ± 0.48	1.57 ± 0.43	0.913
	FEV1 (% predicted)	56.25 ± 10.7	53.9 ± 12.9	0.602
RTX M + 6	SPO2% desaturation	2.63 ± 3.9	8.43 ± 8.6	**0.021**
	FVC in L	1.83 ± 0.53	1.54 ± 0.48	0.127
	FVC(% predicted)	55.4 ± 11.4	44.1 ± 11.6	**0.012**
	FEV1 in L	1.65 ± 0.46	1.4 ± 0.39	0.121
	FEV1(% predicted)	59.04 ± 10.9	47.7 ± 12.7	**0.014**
Rate of change of FVC ^#^		6.08 ± 6.8	−11.03 ± 9.01	**< 0.001**

*: Independent samples T test. #: Rate of change of FVC% predicted from M0 to + 6 M.

There was no significant difference between responder and non-responder groups of patients regarding their age, gender, smoking status, or their radiological findings, however, non-responder group of patients had higher BMI (31.7 ± 5.3 vs. 28.1 ± 8.1, *p* = 0.037) and unidentified antigen exposure (42.9% vs. 6.25%, *p* = 0.031) as illustrated in Table 4. From this data we concluded that BMI (OR 0.804, 95%CI 0.649–0.997) could predict good response to rituximab by using Univariate analysis; as illustrated in Table 5.

**Table 4:** Demographic and radiological patterns of responder and non-responder groups of patients.

	Responders (*n* = 16)	Non-responders (*n* = 14)	*P*
Gender			
Male	1 (6.25)	2 (14.3)	0.586 ^a^
Female	15 (93.75)	12 (85.7)	
Age (mean ± SD) in years	36.9 ± 9.8	43.4 ± 11.3	0.108 ^b^
BMI (mean ± SD) in kg/m^2^	28.1 ± 8.1	31.7 ± 5.3	**0.037** ^b^
Smoking status			
Former	0 (0)	1 (7.1)	0.467 ^a^
Non-smoker	16 (100)	13 (92.9)	
Antigen exposure			
known specific	15 (93.75)	8 (57.1)	**0.031** ^a^
Unknown	1 (6.25)	6 (42.9)	
Antigen type			
Domestic/Birds	15 (93.75)	7 (50)	**0.025** ^c^
Occupational	0 (0)	1 (7.1)	
Not known	1 (6.25)	6 (42.9)	
Centrilobular nodulations			
Present	13 (81.25)	8 (57.1)	0.236 ^a^
Absent	3 (18.75)	6 (42.9)	
Diffuse GGOs			
Present	4 (25)	5 (35.7)	0.694 ^a^
Absent	12 (75)	9 (64.3)	
Mosaicism			
Present	7 (43.75)	4 (28.6)	0.389 ^c^
Absent	9 (56.25)	10 (71.4)	
Traction Bronchiectasis			
Present	1 (6.25)	5 (35.7)	0.072 ^a^
Absent	15 (93.75)	9 (64.3)	
Lobular air trapping			
Present	2 (12.5)	4 (28.6)	0.378 ^a^
Absent	14 (87.5)	10 (71.4)	

a: Fisher’s exact test. b: Independent samples T test. c: Chi square test.

**Table 5:** Univariate analysis of predictors of response for the group of patients who responded to rituximab therapy.

	Sig.	Odds Ratio	95% CI	
			Lower	Upper
Age (years)	0.115	0.942	0.874	1.015
BMI	**0.047**	0.804	0.649	0.997
Dyspnea grade at M0 before the start of Rituimab therapy (by mMRC scale)	0.113	0.251	0.046	1.385
Centrilobular nodulations	0.159	0.308	0.060	1.589
Diffuse GGOs	0.525	1.667	0.346	8.038
Mosaicism	0.392	0.514	0.112	2.359
Traction Bronchiectasis	0.071	8.333	0.835	83.167
Lobular air trapping	0.283	2.8	0.427	18.375

### Adverse events

No cases of mortality were reported in the studied cohort. Lower respiratory tract infections occurred in 3 patients (10%), while upper respiratory tract infections were observed in 2 patients (6.7%). None of the studied patients developed hypogammaglobulinaemia.

## Discussion

To date, the principal treatment strategy for hypersensitivity pneumonitis (HP) involves systemic corticosteroids alongside strict avoidance of the inciting antigen. Although corticosteroids may facilitate initial symptomatic improvement, they do not appear to modify the long-term disease trajectory, presumably due to their limited impact on the underlying immune mechanisms driving HP pathogenesis, leading to its failure as a line of therapy in some HP patients^9^.

The rationale for Rituximab use in HP is based on its ability to induce B-cell depletion systemically by targeting CD20 + lymphocytes, thereby modulating the immune response and potentially halting fibrotic progression. This immunomodulatory effect may contribute to clinical, functional, and radiological stabilization or improvement in patients with refractory disease^10^.

In this study, although a decline in forced vital capacity (FVC) was still observed following Rituximab administration, the rate of decline was considerably attenuated post-treatment. Specifically, the reduction in FVC between baseline (M0) and six months post-therapy (M + 6) was − 0.93%, which was fundamentally less than the pre-treatment decline of − 7.54% observed from M − 6 to M0 (*p* < 0.001). Our results are consistent with those of Ferreira et al. (2020)^7^, who found that Rituximab has the potential to stabilize or even improve lung function in certain patients. They reported a 3% decline in FVC between M0 and M + 6, which was significantly smaller than the 8% decline observed between M − 6 and M0 (*p* = 0.0002). These findings are consistent with the results of our study.

In that same study by Ferreira et al. (2020)^7^, Rituximab was re-administered between M + 6 and M + 12 in 60% of patients, with further slowing of FVC decline over 12 months. The decline observed over the 12-month interval from M0 to M + 12 (− 7% [− 15%; +10%]) was significantly smaller compared to the decline seen during the 6-month period from M − 6 to M0 (− 8% [− 21%; 0%]; *p* = 0.0017). In our study we categorized patients into two groups based on FVC response at M + 6: “responders” (*n* = 16, 53.3%) and “non-responders” (*n* = 14, 46.7%). In Ferreira et al. (2020)^7^’s cohort, patients were divided into two groups: “responders” (*n* = 7, 35%), who showed an improvement in FVC at M + 6, and “non-responders” (*n* = 13, 65%), whose FVC consistently declined compared to M0. In our study the number of responders to rituximab therapy was higher when compared to Ferreira et al. (2020)^7^, this may be attributed to their inclusion of patients with more predominant and extensive fibrotic patterns on radiological imaging, which was not the case in our study.

Earlier data from Keir et al. (2014)^11^ support these observations, describing a cohort of 50 patients with progressive ILD (excluding IPF) treated with Rituximab. Their findings suggested a trend toward better preservation of forced vital capacity (FVC) following rituximab therapy. Specifically, pulmonary function testing performed 6–12 months after rituximab initiation showed a median FVC improvement of 6.7% (*p* < 0.01), in contrast to a median FVC decline of 14.3% during the 6–12 months preceding treatment. No significant differences were observed between responders and non-responders in terms of age, sex, ILD subtype, or radiological patterns such as organizing pneumonia or non-specific interstitial pneumonia. The study included six patients with hypersensitivity pneumonitis (HP), all of whom experienced progressive decline despite intensive conventional immunosuppression, with a median FVC decrease of − 13.3% (range − 8.5% to − 33.3%) in the year prior to rituximab. Following treatment, lung function stabilized or improved in three patients, whereas the remaining three continued to decline and died within four months of receiving rituximab.

Additional analyses of Ferreira et al. (2020)^7^ indicated that no single clinical or demographic variable significantly predicted treatment response. However lack of UIP/NSIP pattern on histopathology was marginally significant (*p* = 0.06). Correspondingly, in our study, no significant differences in age, sex, smoking history, or radiological patterns were observed between responders and non-responders. Notably, non-responders had a significantly higher body mass index (BMI) (31.7 ± 5.3 kg/m² vs. 28.1 ± 8.1 kg/m², *p* = 0.037). Univariate analysis revealed that BMI was a negative predictor of response to Rituximab (OR 0.804, 95% CI: 0.649–0.997).

Our findings are in accordance with those of Iannone et al. (2015)^12^, who reported diminished treatment efficacy in obese rheumatoid arthritis patients receiving non-anti-TNF agents, primarily Rituximab. However, evidence from Ottaviani et al. (2015)^13^ showed comparable long-term outcomes in obese and non-obese patients. The pro-inflammatory environment linked to obesity could account for the differential response. It is well known that adipokines and cytokines released by adipose tissue can influence the response to treatment by causing systemic inflammation^14^. Notably, worse outcomes have been linked to elevated levels of leptin and adiponectin during exacerbations of idiopathic pulmonary fibrosis^15^.

In a recent study by Adams et al. (2023)^16^, 71.4% (*n* = 15) of patients were classified as responders to rituximab therapy. The responder group was further subdivided into stable responders (RSP-stable, *n* = 9) and improving responders (RSP-improv, *n* = 6). After six months, half of the improving responders demonstrated reductions in ground-glass opacities and fine fibrosis on high-resolution computed tomography (HRCT), while the remaining half showed no radiological change. All stable responders exhibited unchanged interstitial lung disease (ILD) features on HRCT. In contrast, among the non-responders, five out of six showed worsening ILD features, with only one patient showing no radiological progression.

### Conclusion

In our study we found that Rituximab appears to be generally safe and well-tolerated among our patients with treatment refractory non-fibrotic HP with elevated BAL lymphocytosis.

### Limitations

Our study has certain limitations, first we did not incorporate DLCO measurements as an assessment tool to be associated with spirometric findings to have a good comprehensive pulmonary function assessment of our patients that would provide a deeper insight into treatment effects of rituximab. Second, a larger study population with multicenter data could have better identified some predictors of response to rituximab therapy in treatment refractory HP patients. This study presents descriptive comparisons of FVC before and after rituximab therapy. Potential confounding factors, such as disease duration since the onset of hypersensitivity pneumonitis and prior steroid dose, were not adjusted for in the analysis, which should be considered a limitation. In addition optimal dosing strategy, the need for additional rituximab doses and longer follow up periods of those patients are essentially needed to evaluate clinical, functional and radiological response to rituximab therapy together with any upcoming long term adverse effects of rituximab therapy. Lastly, this observational, one-arm study limits definitive conclusions about treatment effects.

## Data Availability

The datasets generated and/or analyzed during the current study are available from the corresponding author upon reasonable request.
